# Brain-derived neurotrophic factor produced long-term synaptic enhancement in the anterior cingulate cortex of adult mice

**DOI:** 10.1186/s13041-021-00853-z

**Published:** 2021-09-15

**Authors:** Hui-Hui Miao, Zhuang Miao, Ji-Gang Pan, Xu-Hui Li, Min Zhuo

**Affiliations:** 1 Department of Anesthesiology, Beijing Shijitan Hospital, Capital Medical University, Beijing, 100038 People’s Republic of China; 2 Department of Physiology, Faculty of Medicine, University of Toronto, Medical Science Building, 1 King’s College Circle, Toronto, ON M5S 1A8 Canada; 3 Center for Neuron and Disease, Frontier Institute of Science and Technology, Xi’an Jiaotong University, Xi’an, 710049 Shaanxi People’s Republic of China; 4 Institute for Brain Research, QingDao International Academician Park, Qing Dao, Shandong People’s Republic of China

**Keywords:** Brain-derived neurotrophic factor, Anterior cingulate cortex, Adenylyl cyclase subtype 1, AMPA, LTP

## Abstract

Previous studies have demonstrated that brain-derived neurotrophic factor (BDNF) is one of the diffusible messengers for enhancing synaptic transmission in the hippocampus. Less information is available about the possible roles of BDNF in the anterior cingulate cortex (ACC). In the present study, we used 64-electrode array field recording system to investigate the effect of BDNF on ACC excitatory transmission. We found that BDNF enhanced synaptic responses in a dose-dependent manner in the ACC in C57/BL6 mice. The enhancement was long-lasting, and persisted for at least 3 h. In addition to the enhancement, BDNF also recruited inactive synaptic responses in the ACC. Bath application of the tropomyosin receptor kinase B (TrkB) receptor antagonist K252a blocked BDNF-induced enhancement. L-type voltage-gated calcium channels (L-VGCC), metabotropic glutamate receptors (mGluRs), but not NMDA receptors were required for BDNF-produced enhancement. Moreover, calcium-stimulated adenylyl cyclase subtype 1 (AC1) but not AC8 was essential for the enhancement. A selective AC1 inhibitor NB001 completely blocked the enhancement. Furthermore, BDNF-produced enhancement occluded theta burst stimulation (TBS) induced long-term potentiation (LTP), suggesting that they may share similar signaling mechanisms. Finally, the expression of BDNF-induced enhancement depends on postsynaptic incorporation of calcium-permeable AMPA receptors (CP-AMPARs) and protein kinase Mζ (PKMζ). Our results demonstrate that cortical BDNF may contribute to synaptic potentiation in the ACC.

## Background

Brain-derived neurotrophic factor (BDNF) is a member of the neurotrophin family which plays a diverse, and broad, role in regulating neuronal structure and function in the central nervous system [1]. Cumulative data has demonstrated that BDNF is crucially involved in synaptic plasticity in the adult brain [2]. Application of BDNF can trigger a long-lasting increase in synaptic transmission in the hippocampus, visual cortex, insular cortex, and spinal dorsal horn [3–7], although some of these enhancements are found to be activity-dependent, or unreliable. Most of the previous studies have focused on the hippocampus. Less information is available about the BDNF related synaptic enhancement in the anterior cingulate cortex (ACC).

ACC is a key cortical area for processing painful and emotional information [8–11]. Adult synapses in the ACC are highly plastic, and both presynaptic release of glutamate and postsynaptic excitatory responses can undergo long-term potentiation (LTP) [8, 12]. The molecular mechanism of ACC LTP has been recently investigated [12–14]. Inhibition or genetic deletion of key molecules required for triggering LTP produces analgesic effects in animal models of chronic pain as well as emotional fear, anxiety, and depression [13, 14]. Several studies have indicated that ACC BDNF can be regulated in an activity-dependent manner, and its alternation may contribute to chronic pain, depression, and other mental disorders [15–18]. For example, Thibault et al. reported that injections of recombinant BDNF into the ACC triggered neuronal hyperexcitability and cold allodynia [15]. Tropomyosin receptor kinase B (TrkB) receptor and ERK phosphorylation were involved in BDNF induced effects [15]. However, the mechanism of BDNF induced synaptic enhancement is largely unknown. In the present study, we aimed to study the effect of BDNF on synaptic transmission in ACC by using a 64-channel multielectrode (MED64) recording system. Furthermore, calcium-stimulated adenylyl cyclase subtype 1(AC1), calcium-permeable AMPA receptors (CP-AMPARs), and protein kinase Mζ (PKMζ) might be involved in BDNF induced synaptic enhancement in ACC.

## Methods

### Animals

Adult (8–12 weeks old) male C57BL/6 mice were purchased from Charles River. *AC1* KO and *AC8* KO mice with the C57BL/6 background were obtained from Dr. Daniel R. Storm (University of Washington, Seattle, WA). All animals were housed under a 12 h light/dark cycle with food and water provided ad libitum. All works were conducted according to the policy and regulation for the care and use of laboratory animals approved by Institutional Animal Care and Use Committee at University of Toronto.

### Brain slice preparation

Adult male mice were anesthetized with 1–2% isoflurane and the whole brain was quickly removed and transferred to ice-cold oxygenated (95% O_2_ and 5% CO_2_) artificial cerebrospinal fluid (ACSF) containing (in mM) 124 NaCl, 2.5 KCl, 2 CaCl_2_, 2 MgSO_4_, 25 NaHCO_3_, 1 NaH_2_PO_4_ and 10 glucose, pH 7.3–7.4. Three coronal brain slices (300 μm), after the corpus callosum meets and contains ACC, were cut using a vibratome (Leica VT 1200S). The slices were placed in a submerged recovery chamber with oxygenated (95% O_2_ and 5% CO_2_) ACSF at room temperature for at least 1 h.

### The 64 multi-electrode arrays

The multielectrode array (MEA) system used in the current study was MED64 (Alpha-Med Sciences, Japan). The procedures for preparation of the MED64 system were similar to those described previously [19]. The MED64 probe (MED-P515A, Panasonic, Japan) were arranged in an 8 × 8 pattern, with an interpolar distance of 150 μm. The surface of the MED64 probe was treated with 0.1% polyethyleneimine (Sigma, St. Louis, MO; P-3143) in 25 mmol/L borate buffer (pH 8.4) overnight at room temperature before experiments. Then the surface of probe was rinsed three times with sterile distilled water [20, 21].

### Field potential recording in adult ACC slices

After recovery, one ACC slice was placed in a MED64 probe covering most of the 64 electrodes. Slices were kept in the recording chamber for 1 h after transfer and perfused with oxygenated (95% O_2_ and 5% CO_2_) ACSF at 28 °C and maintained at a 3–4 ml/min flow rate. The slice was positioned on the MED64 probe that the different layers of the ACC were entirely covered by the whole array of the electrodes, and then a fine-mesh anchor was placed on the slice to ensure its stabilization during the experiments [22]. One of the channels located in the layer V of the ACC was chosen as the stimulation site, from which the best synaptic responses can be induced in the surrounding recording channels. Bipolar constant current pulse stimulation was applied to the stimulation channel and the intensity was adjusted so that 40–60% of maximal field excitatory postsynaptic potential (fEPSP) was elicited in the channels closest to the stimulation site. The channels with fEPSPs were considered as active channels and their fEPSPs responses were sampled every 2 min. The parameter of “slope” indicated the averaged slope of each fEPSP recorded by activated channels. Then, the drug of BDNF was applied for 30 min before washout. After BDNF infusion, the test stimulus was repeatedly delivered for at least 3 h to allow long-term monitoring. In another set of experiments, paired-pulse facilitation (PPF) with the interpulse interval at 50 ms was recorded to determine the locus of synaptic transmission expression. The ratio of the slope of the second response to the slope of the first response was calculated and averaged. In the occlusion test, a theta-burst stimulation (TBS) (five trains of bursts with four pulses at 100 Hz at 200 ms interval; repeated five times at intervals of 10 s) was applied to the same stimulation channel. In most experiments, 6–8 channels near the stimulation site were selected for data analysis.

### Drugs

Drugs were prepared as stock solutions for frozen aliquots at − 20 °C. They were diluted from the stock solution to the final desired concentration in the ACSF before being applied to the perfusion solution. BDNF (1, 20, 50 ng/ml), nimodipine (10 μM), d (−)-2-amino-5-phosphonopentanoic acid (AP-5, 100 μM), (+)-α-methyl-4-carboxylphenlyglycine (MCPG, 500 μM), K252a (200 nM), NASPM (50 μM), ZIP (5 μM) and NB001 (20 μM) were used in the current study. Drugs were infused during the period of the horizontal bar on the graphs. NB001 was provided by NeoBrain Pharmac Inc (Canada). BDNF, nimodipine, MCPG, K252a, ZIP was purchased from Tocris Bioscience. AP-5, NASPM were purchased from Hello Bio.

### Data analysis

MED64 Mobius was used for data acquisition and analysis. All data were presented as means ± SEM. The percentages of the fEPSP slopes were normalized by the averaged value of the baseline. The synaptic enhancement levels used in histograms are averaged fEPSP slope value of the last 20 min of the experiment. Statistical comparisons were performed using two-tail paired or unpaired Student’s *t*-test, one-way ANOVA by GraphPad Prism 6.0.

## Results

### BDNF induced long-term synaptic enhancement in the ACC

**Fig. 1:**
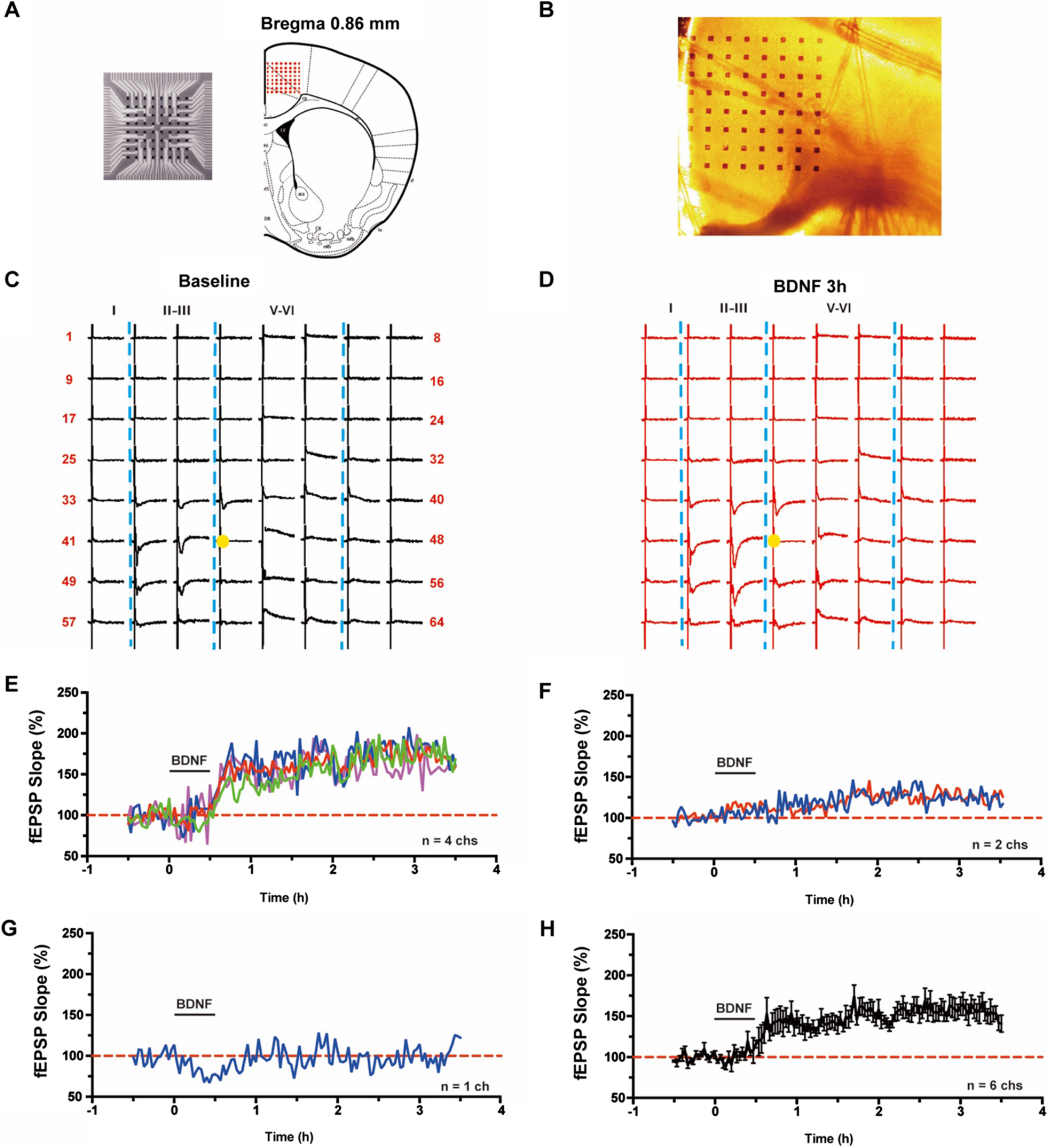
Spatial distribution of BDNF produced synaptic enhancement in the ACC obtaining with multichannel field potential recording. **A** Schematic diagram and the microphotograph showed the scale of the MED-64 probe (left), location of MED-64 probe on the ACC slice (right). **B** One example microscopy photograph of the location of ACC slice and MED-64 probe. **C** A general view of the spatial distribution of the potentials in all active channels within different layers (vertical lines) of one ACC slice at baseline period. **D** An overview of the spatial distribution of the evoked potentials in all active channels across different layers (vertical lines) of one ACC slice at 3 h after BDNF perfusion. **E**–**G** Individual data of changes of the fEPSP slopes with three types of plasticity from one ACC slice of applying BDNF: **E** Long-lasting synaptic enhancement showing channels; **F** short-time synaptic enhancement showing channels; **G** No enhancement showing channels. **H** Summarized date showed fEPSP slopes from channels with synaptic enhancement after the application of BDNF in one ACC slice

**Fig. 2:**
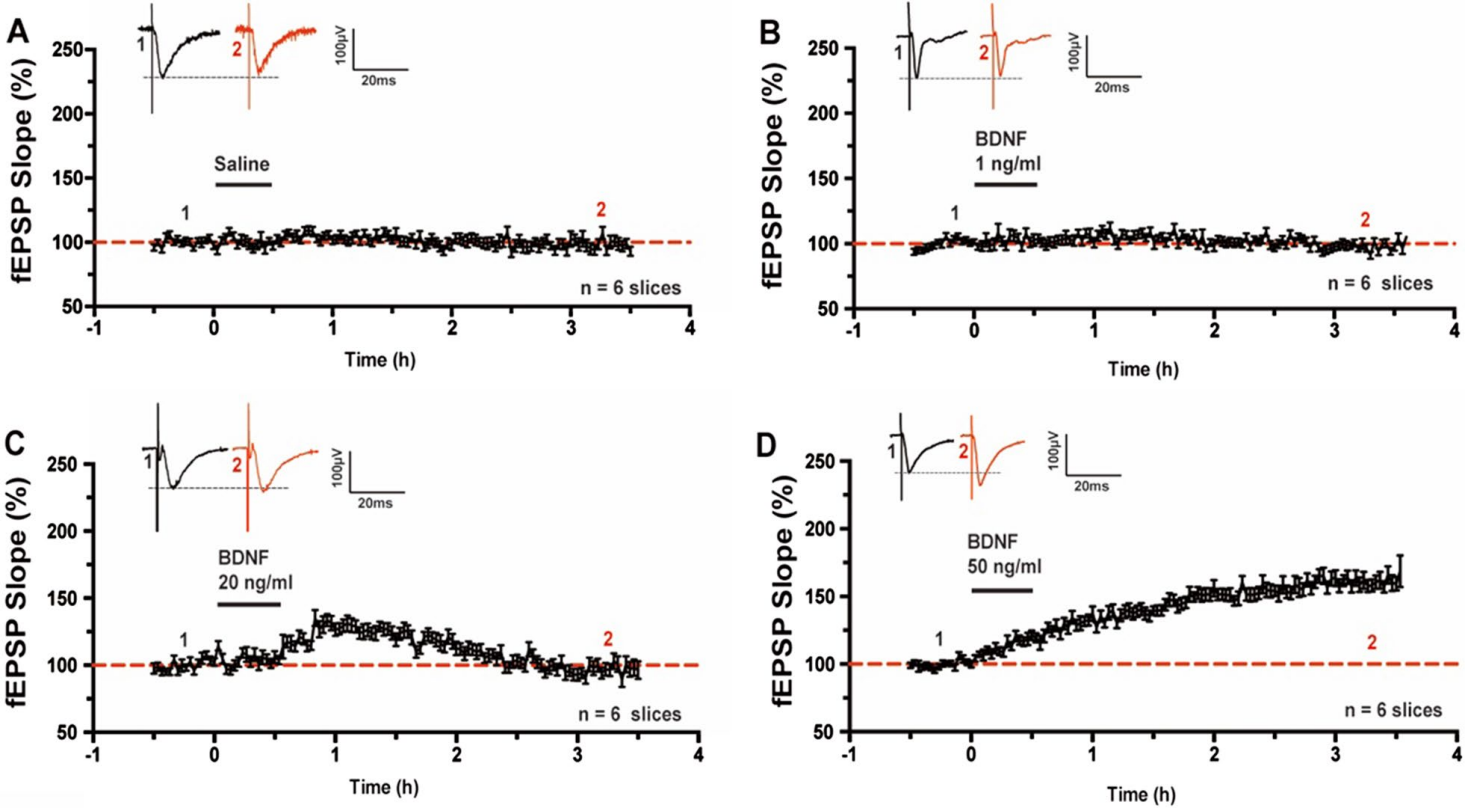
BDNF dose-dependently produced chemical synaptic enhancement in the ACC. **A** Pooled fEPSP slopes illustrated that 0.9% saline did not change the fEPSP slope in the ACC. **B** Pooled fEPSP slopes illustrated that 1 ng/ml BDNF failed to produce synaptic enhancement in the ACC. **C** Pooled fEPSP slopes illustrated that the time course of 20 ng/ml BDNF produced short-time synaptic enhancement in the ACC. **D** Pooled fEPSP slopes illustrated that the time course of 50 ng/ml BDNF produced long-lasting synaptic enhancement in the ACC

**Fig. 3:**
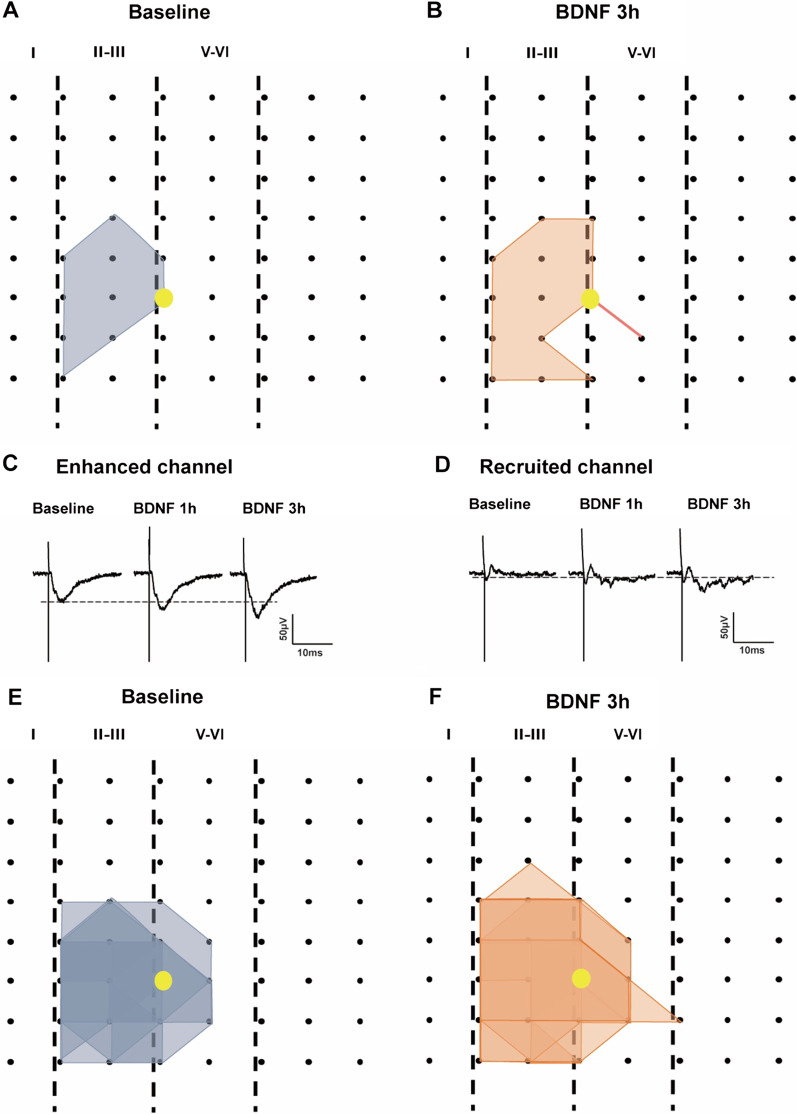
BDNF enhanced the network propagation of synaptic responses in the ACC. **A**, **B** Sample polygonal diagram showed the distribution of activated channels in the baseline state (gray) and BDNF recruited channels (orange). The yellow circle indicated the stimulation site. **C**, **D** The sample traces showed the long-lasting synaptic enhancement and recruited channel at baseline and 1 h, 3 h after BDNF application. **E**, **F** Superimposed polygonal diagrams of the activated channels in the baseline state (gray) and the enlarged area after application of BDNF (orange). Black dots represented the 64 channels in the MED64. Vertical lines indicated the layers in the ACC slice

In the present study, we used the 64-channel multielectrode array, MED64 [14, 20], to examine if BDNF might affect excitatory synaptic transmission in the ACC. One representative example recording was illustrated in Fig. 1. The relative location of the 8 × 8 square-shaped MED64 probe within the ACC slice was shown in Fig. 1A. The light microscope photograph of the location of the MED64 probe electrodes within the ACC slice was showed in Fig. 1B. One channel in the deep layer V of ACC was chosen as the stimulation site (Fig. 1C, D) and fEPSPs were recorded from the other 63 channels around the stimulation site. Activated channels with fEPSPs around the stimulation channel expressed from the superficial to deep layers of ACC during baseline period (Fig. 1C) and 3 h after BDNF perfusion (Fig. 1D) were shown. After 30 min stable baseline recording, BDNF (50 ng/ml) was applied for 30 min and then washed out. We found that BDNF induced synaptic enhancement in the ACC slice with four active channels showing long-term enhancement lasting over 3 h (Fig. 1E) and two active channels showing a short-term enhancement lasting less than 2 h (Fig. 1F), with one channel remaining unchanged throughout the entire recording period (Fig. 1G). The averaged data from the six active channels was plotted in Fig. 1H. We then tested the dose–response of BDNF effects on synaptic transmission in the ACC (Fig. 2). As shown in Fig. 2A and B, 0.9% saline or 1 ng/ml BDNF for the same duration did not change the fEPSP slope in the ACC (Saline: 98.2 ± 3.1% of baseline, n = 6 slices/5 mice; 1 ng/ml BDNF: 97.3 ± 2.1% of baseline, n = 6 slices/6 mice). However, application of 20 ng/ml BDNF induced a short-term enhancement in the ACC, which lasted less than 2 h (124.9 ± 3.4% of baseline at 1 h after BDNF perfusion; 97.3 ± 3.8% of baseline at 3 h after BDNF perfusion, n = 6 slices/6 mice, Fig. 2C). Finally, we found that 50 ng/ml BDNF induced a long-term enhancement in the ACC that lasted over 3 h (159.8 ± 4.5% of baseline, n = 6 slices/6 mice, Fig. 2D). This data suggests that BDNF induces a dose-dependent synaptic enhancement in the ACC.

### Spatial distribution and recruitment of BDNF induced synaptic responses

Next, we mapped the spatial distribution of the active responses in the ACC before, and after, BDNF perfusion using the previous method [20]. In one typical sample slice, as shown in the Fig. 3A, the distribution of all observed activated-channels during baseline period was displayed by a polygonal diagram on a grid representing the channels (gray). Application of BDNF (50 ng/ml) significantly enhanced the spatial distribution of active responses in the ACC (Fig. 3B). The enlarged area was observed in both the deep, and superficial, layers in the ACC. Our previous studies reported that recruitment of synaptic responses in the TBS induced L-LTP [14, 20]. The recruited channels were also observed by application of BDNF. The sample traces and recruited channels were shown in Fig. 3C and D, the recruited channel showing no fEPSP at baseline, but with increased potentiation at 1 h and 3 h after BDNF application. Plotted data from six slices for the spatial distribution of the active responses before and after BDNF perfusion were shown in Fig. 3E and F. These results suggest that BDNF application was also effective for spatial changes of synaptic enhancement in the ACC.

### Effects of BDNF application on paired-pulse facilitation (PPF)

**Fig. 4:**
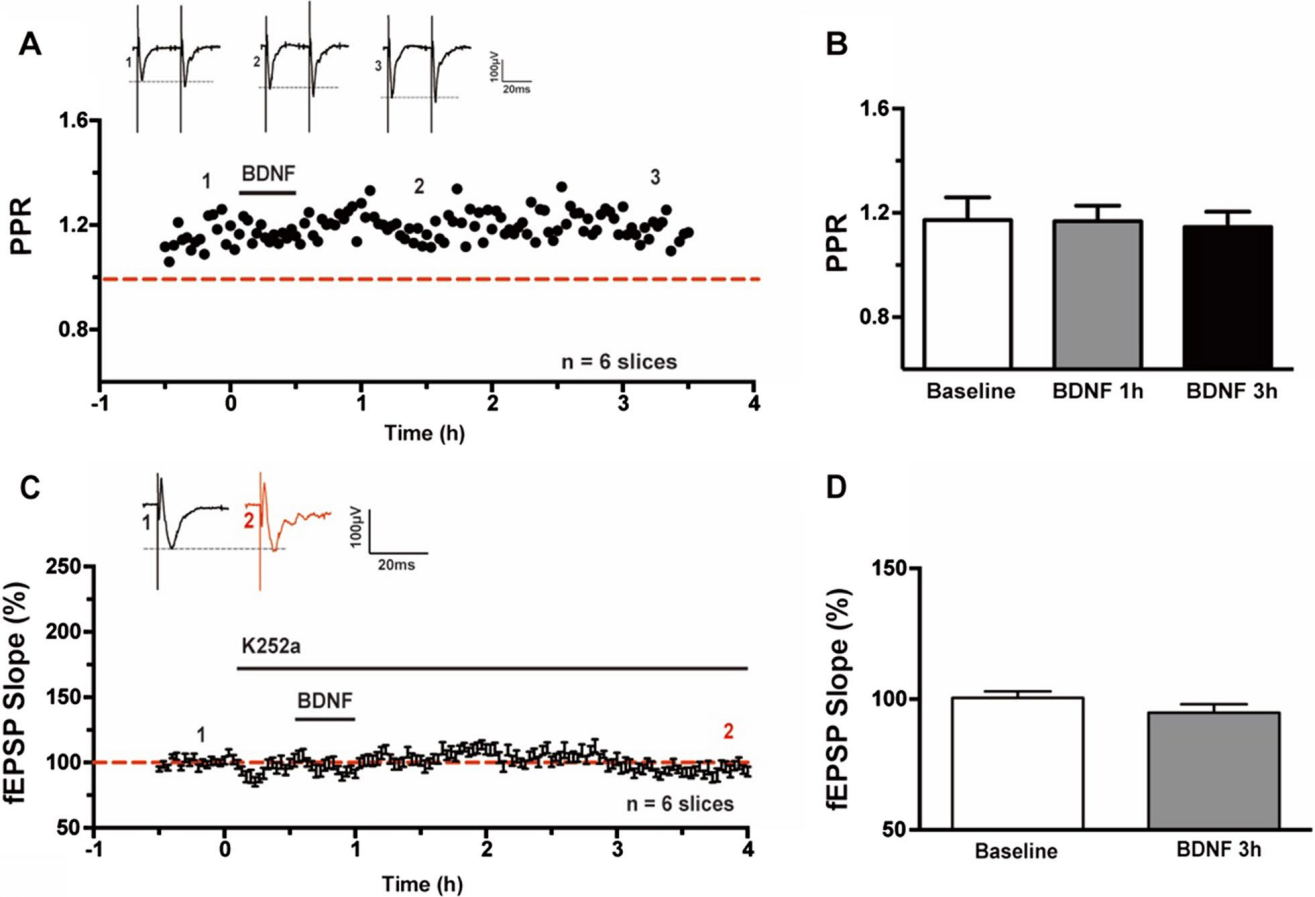
Effects of BDNF produced synaptic enhancement on PPF and TrkB receptor in the ACC. **A** Averaged data showed the PPF was not changed before and after BDNF perfusion. **B** Bar histogram showing the pooled data of PPR from six slices of six mice. **C** Bath applied K252a (200 nM) 30 min before BDNF blocked the enhancement in the ACC slices. **D** Bar histogram showing the pooled data of fEPSP Slope (%) of baseline and 3 h after BDNF application with K252a from six slices of six mice

PPF is often used for measuring presynaptic function. To investigate the locus of BDNF induced synaptic enhancement expression, we recorded the PPF with 50 ms interval during the baseline and at 1 h, 3 h after BDNF application. As shown in Fig. 4A and B, the PPF was not significant changed (1.17 ± 0.09 at baseline; 1.17 ± 0.06 at 1 h and 1.15 ± 0.06 at 3 h, n = 6 slices/6 mice, *P* = 0.91, one-way ANOVA), indicating that the BDNF produced synaptic transmission was most likely postsynaptic.

### BDNF induced synaptic enhancement was blocked by inhibition of TrkB receptor

To test whether the effect of BDNF on ACC field potentials are receptor-specific, we explored the role of TrkB receptor activation in BDNF induced synaptic enhancement pharmacologically, as shown in Fig. 4C, K252a (200 nM) was bath applied 30 min before BDNF and throughout the recording period, then BDNF induced enhancement was completely blocked. The fEPSP slope (%) of baseline and 3 h after BDNF application was not significant changed (100.5 ± 0.5% vs. 94.8 ± 3.3%; n = 6 slices/6 mice, *P* = 0.10, paired *t*-test, Fig. 4D).

### BDNF induced synaptic enhancement and TBS-LTP occluded each other in the ACC

**Fig. 5:**
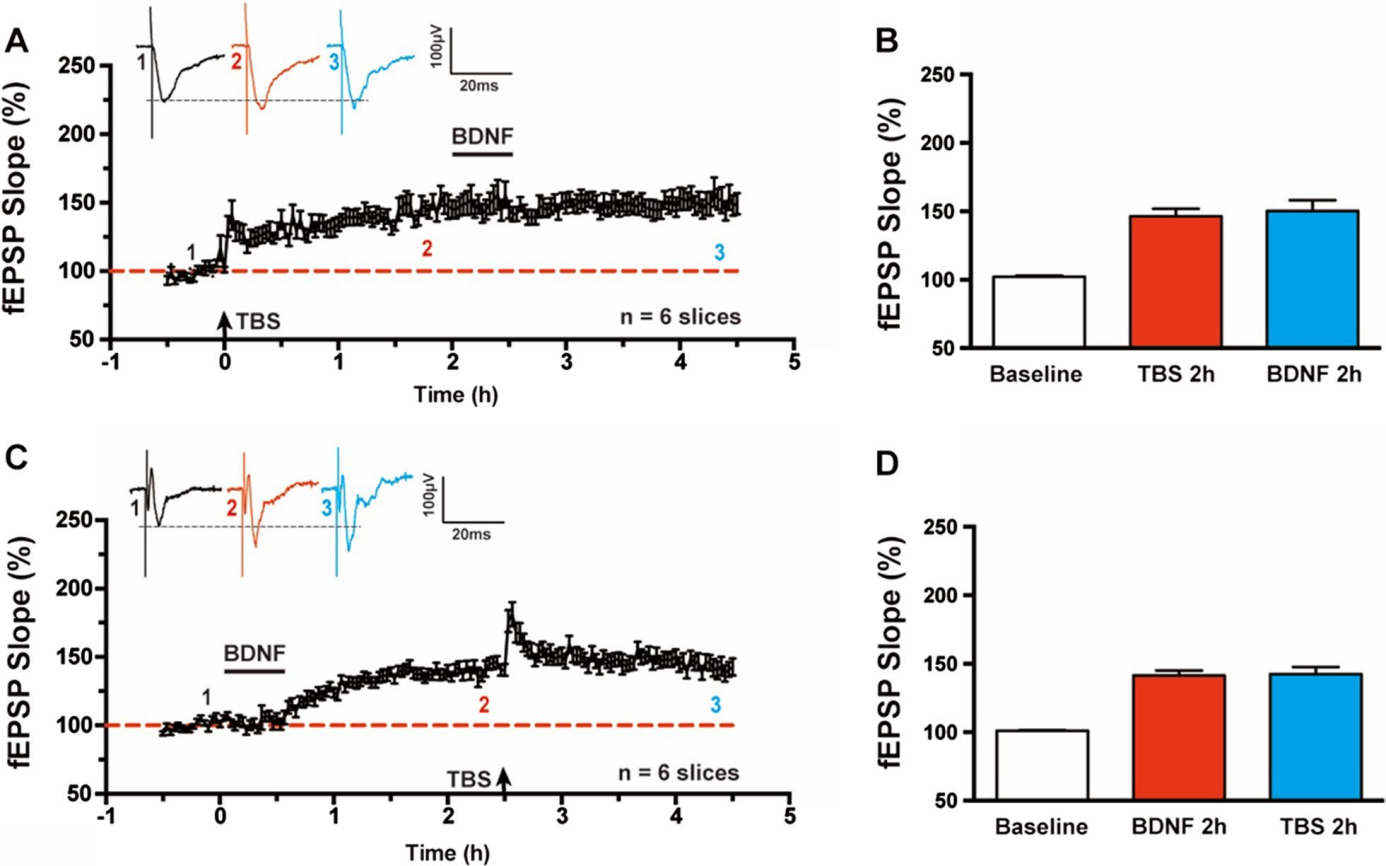
BDNF produced synaptic enhancement and TBS-LTP occluded each other in the ACC. **A** BDNF produced synaptic enhancement was occluded by TBS-LTP at 2 h after theta-burst stimulation in the ACC. **B** Bar histogram showing the pooled data of fEPSP Slope (%) of baseline, 2 h after TBS and 2 h after BDNF application from six slices of six mice. **C** TBS-LTP was occluded by BDNF induced enhancement at 2 h after application in the ACC. **D** Bar histogram showing the pooled data of fEPSP Slope (%) of baseline, 2 h after BDNF application and 2 h after TBS from six slices of six mice

To test whether the BDNF induced synaptic enhancement might share common cellular mechanisms with TBS induced LTP, we investigated the ability of each to occlude subsequent induction of the other form. BDNF was applied at 2 h after LTP induction by TBS (Fig. 5A). We found that TBS-LTP occluded the enhancement of synaptic transmission by BDNF application. The fEPSP slope at 2 h after TBS-LTP induction was 146.2 ± 5.6% and 150.1 ± 7.8% at 2 h after subsequent BDNF perfusion (n = 6 slices/6 mice, *P* = 0.34, paired *t*-test, Fig. 5B). Oppositely, BDNF induced synaptic enhancement also attenuated the subsequent potentiation by TBS (Fig. 5C). The fEPSP slope at 2 h after BDNF perfusion was 141.3 ± 3.7% and 142.4 ± 5.2% at 2 h after subsequent TBS-LTP induction (n = 6 slices/6 mice, *P* = 0.81, paired *t*-test, Fig. 5D). Taken together, this data suggests that these two forms of synaptic enhancement in the ACC may involve some similar mechanisms.

### L-VGCC, mGluR but not NMDA receptors were required for BDNF induced synaptic enhancement

**Fig. 6:**
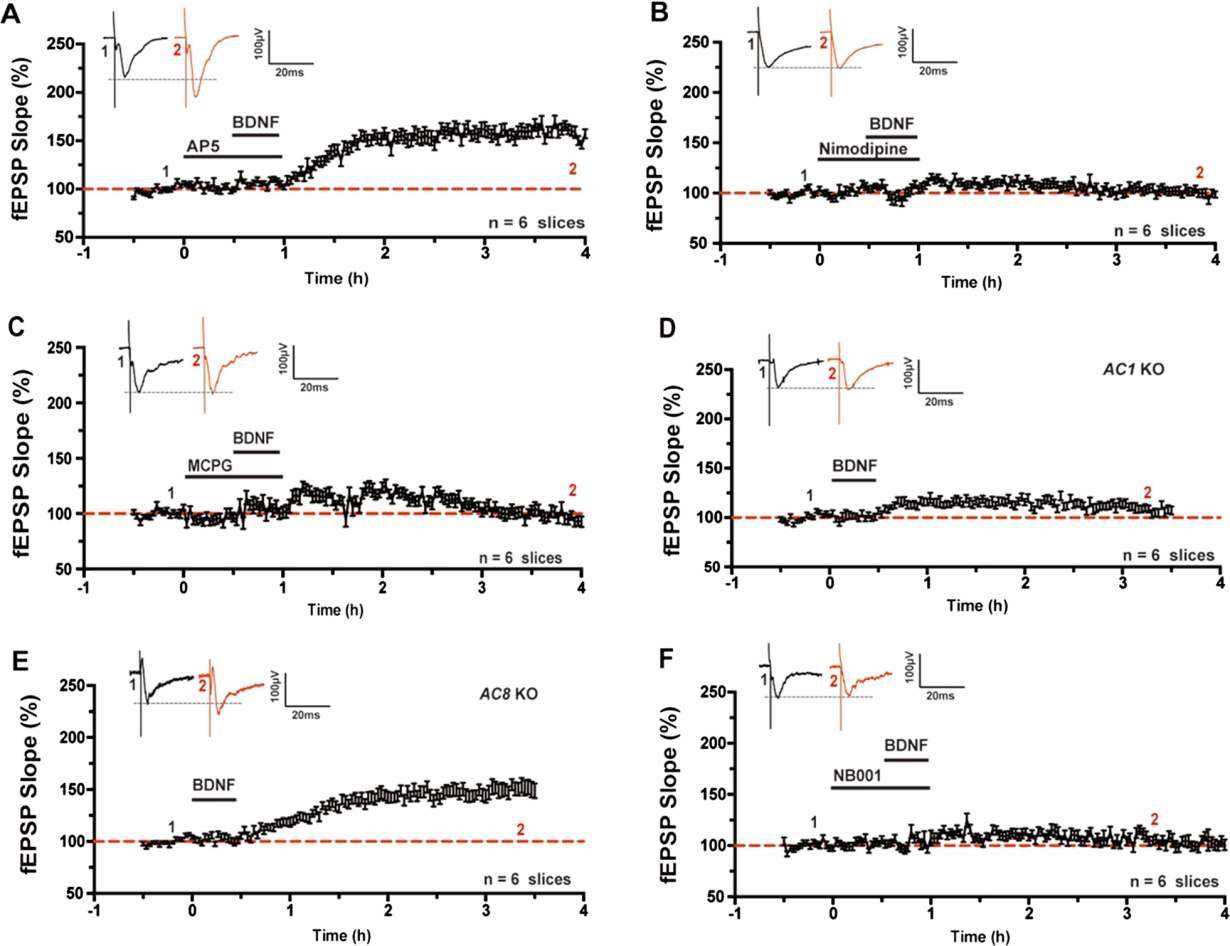
Mechanism of BDNF produced synaptic enhancement in the ACC. **A** NMDA receptor antagonist AP5(100 μM) had no effect on BDNF produced synaptic enhancement in the ACC. **B** L-VGCC blocker nimodipine (10 μM) blocked BDNF induced synaptic enhancement in the ACC. **C** mGluR antagonist MCPG (500 μM) blocked BDNF produced synaptic enhancement in the ACC. **D** In AC1 KO mice, BDNF induced enhancement was attenuated in the ACC slices. **E** In AC8 KO mice, BDNF still induced synaptic enhancement in the ACC. **F** AC1 inhibitor NB001 (20 μM) blocked BDNF produced synaptic enhancement in the ACC

Previous research had shown that effects on TBS induced synaptic enhancement in the ACC requires the activation of NMDARs. Hence, we investigated whether BDNF induced synaptic enhancement in the ACC was dependent on the activation of the NMDA receptor. The NMDA receptor antagonist AP-5 (100 μM) was bath-applied 30 min before the application of BDNF and then were washed out together. As shown in Fig. 6A, the fEPSP slope was increased and lasted for 3 h (159.1 ± 4.5%, n = 6 slices/6 mice), indicating that BDNF produced NMDA receptor independent synaptic enhancement in the ACC. Next, to determine any involvement of L-VGCC in BDNF induced synaptic enhancement, we perfused an ACC slice with nimodipine (a selective L-type voltage-gated calcium channel blocker, 10 μM) and found an effective attenuation of synaptic transmission (100.2 ± 3.3%, n = 6 slices/6 mice) (Fig. 6B). Additionally, we used an mGluR antagonist, MCPG (500 μM) to test the role of metabotropic glutamate receptor in BDNF produced synaptic enhancement. The MCPG significantly blocked BDNF induced synaptic enhancement (97.0 ± 4.0%, n = 6 slices/6 mice) (Fig. 6C). These results suggest that L-VGCC and mGluR, but not NMDA receptors, are important for BDNF produced synaptic enhancement in the ACC.

### AC1 but not AC8 were required for BDNF induced synaptic enhancement

In the ACC, TBS induced postsynaptic LTP requires activation of Ca^2+^/calmodulin-stimulated adenylyl cyclase subtype 1(AC1), but not AC8 [8]. We tested whether AC1 or AC8 was required for BDNF produced synaptic enhancement, and found that in genetic knockout AC1 mice*,* BDNF perfusion did not affect synaptic transmission in the ACC (107.5 ± 3.5%, n = 6 slices/5 mice) (Fig. 6D). Conversely, BDNF still produced synaptic enhancement in AC8 KO mice (150.8 ± 6.1%, n = 6 slices/5 mice) (Fig. 6E). To further test the role of AC1, we used a selective AC1 inhibitor NB001 (20 μM) 30 min before BDNF application, BDNF induced synaptic enhancement was attenuated by NB001 in the ACC (103.1 ± 3.8%, n = 6 slices/6 mice) (Fig. 6F). Taken together, this data suggests that AC1 is essential for BDNF produced synaptic enhancement in the ACC.

### CP-AMPARs and PKMζ were involved in BDNF induced synaptic enhancement

**Fig. 7:**
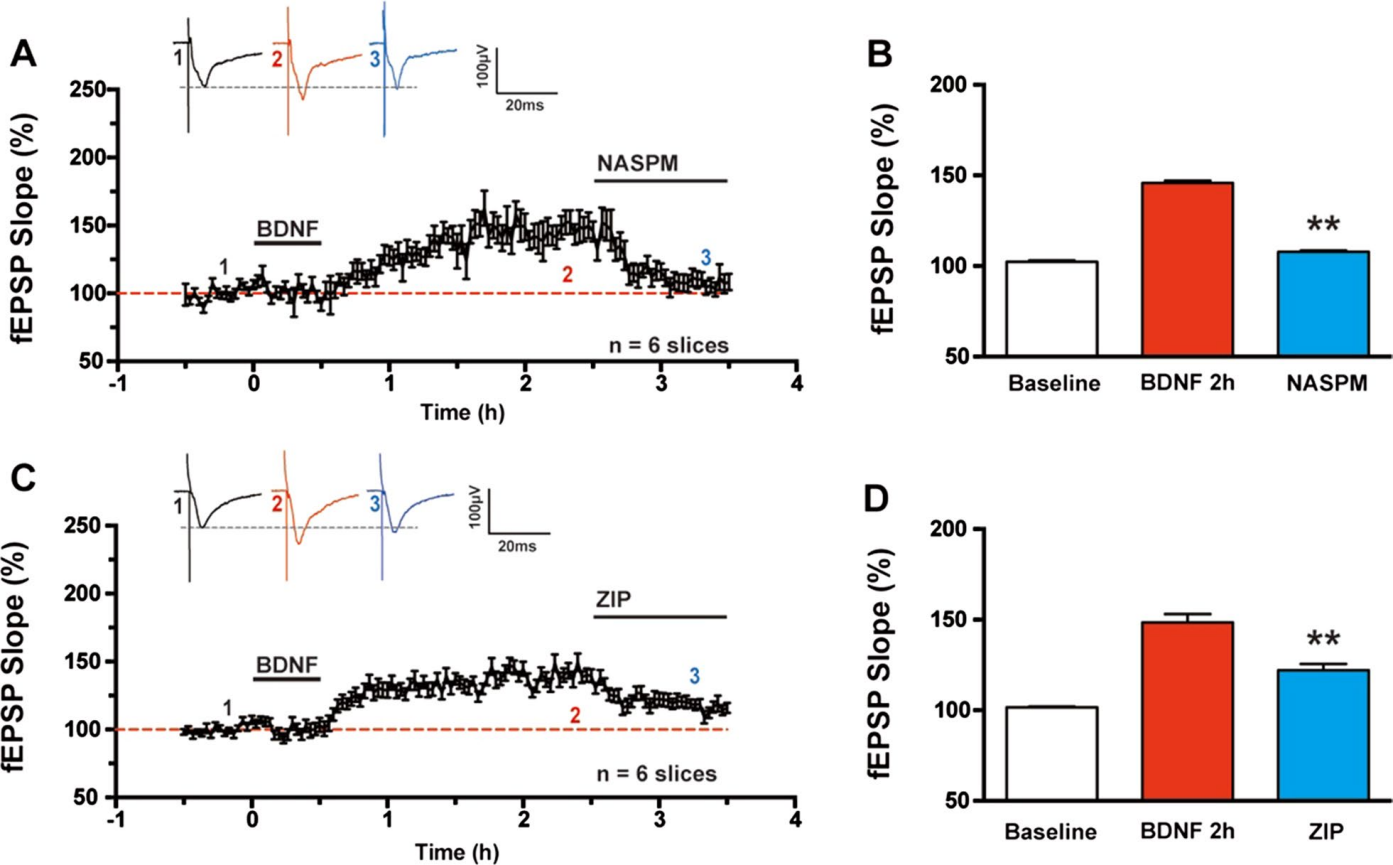
Roles of CP-AMPARs and PKMζ in the expression of BDNF produced synaptic enhancement in the ACC. **A** Bath applied Ca^2+^-permeable AMPA antagonist NASPM (50 μM) at 2 h after BDNF perfusion abolished synaptic potentiation in the ACC. **B** Bar histogram showing the pooled data of fEPSP Slope (%) of NASPM application after BDNF perfusion from six slices of six mice. **C** Bath applied PKM**ζ** inhibitory peptide ZIP (5 μM) at 2 h after BDNF perfusion also reduced the enhancement in the ACC. **D** Bar histogram showing the pooled data of fEPSP Slope (%) of ZIP application after BDNF perfusion from six slices of six mice. **p < 0.01 compared with BDNF induced synaptic enhancement, error bars indicated SEM

In the ACC, changes in postsynaptic AMPARs contribute to the expression of TBS induced L-LTP, and the application of a CP-AMPAR antagonist 3 h after induction reduced synaptic potentiation [13]. To follow, we then tested whether CP-AMPAR affected BDNF induced synaptic enhancement in the ACC. As expected, the perfusion of CP-AMPAR antagonist NASPM (50 μM) 2 h after BDNF perfusion abolished BDNF induced synaptic enhancement (Fig. 7A), the fEPSP slope changed from 147.2 ± 7.1% at 2 h after BDNF perfusion to 108.0 ± 4.8% 1 h after NASPM application (n = 6 slices/6 mice, *P* < 0.01, paired *t*-test, Fig. 7B).

Previous studies suggested that PKMζ was required to maintain persistent synaptic potentiation in the ACC [23]. We also assessed whether PKMζ was involved in BDNF induced synaptic enhancement. The selective PKMζ inhibitor, ζ-pseudosubstrate inhibitory peptide (ZIP, 5 μM), erased synaptic enhancement 2 h after BDNF perfusion in the ACC (Fig. 7C), the fEPSP slope changed from 148.4 ± 4.6% at 2 h after BDNF application to 117.0 ± 2.6% 1 h after ZIP perfusion (n = 6 slices/6 mice, *P* < 0.01, paired *t*-test, Fig. 7D). Therefore, both CP-AMPAR and PKMζ are required for BDNF induced synaptic enhancement.

## Discussion

**Fig. 8:**
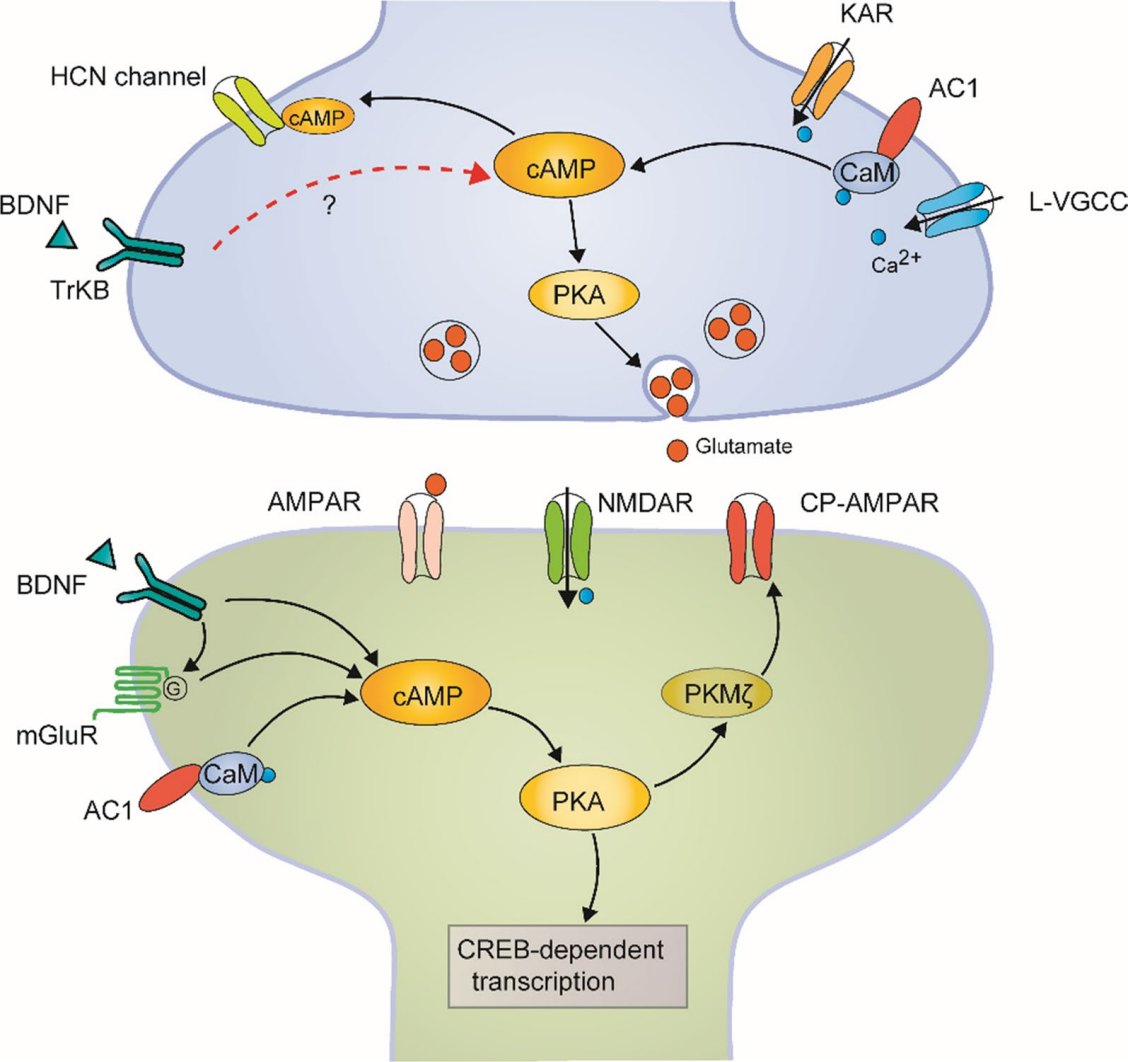
The proposed model explains synaptic mechanisms of BDNF-induced chemical LTP in the ACC. A synaptic model for BDNF-induced LTP. BDNF in the ACC enhanced the synaptic transmission through a postsynaptic mechanism by the activation of TrkB receptor and Ca^2+^-dependent AC1 signal pathway. mGluR, CP-AMPAR, and PKMζ are also involved in the BDNF-induced LTP in the ACC. BNDF likely causes the potentiation by enhancing presynaptic release of glutamate, although we cannot completely rule out the possibility of presynaptic action

In the present study, we demonstrate that bath application of BDNF produces synaptic enhancement in the ACC of adult mice. The BDNF induced enhancement is post-synaptically expressed and prevented by TrkB receptor blocker. BDNF induced synaptic enhancement and TBS-LTP occlude each other in the ACC. Activation of L-VGCC, mGluRs, but not NMDA receptors, were involved in BDNF induced synaptic enhancement and the genetic deletion of AC1, but not AC8, impaired BDNF induced synaptic enhancement. Using the AC1 inhibitor NB001 also blocked BDNF produced synaptic enhancement. CP-AMPARs and PKMζ were involved in BDNF induced synaptic enhancement. Although the functional significance of this BDNF induced potentiation remains to be investigated in future, it is likely that BDNF may contribute to chronic pain, emotional anxiety, fear, or depression in the ACC (Fig. 8).

### BDNF induced synaptic enhancement in the ACC

Previous investigation of the effect of BDNF on central synaptic transmission were mainly focused on the hippocampus, visual cortex, and spinal dorsal horn [4, 5, 7]. The impairment of synaptic transmission in BDNF-knockout mice could be rescued by acute application of BDNF [24]. In the ACC, injections of recombinant BDNF or a viral vector synthesizing BDNF triggered long-term potentiation and sustained pain hypersensitivity [15]. In the present study, we found that exogenous application of dose-dependent BDNF increased synaptic transmission in the ACC slices. Moreover, BDNF-TrkB signaling was required for this chemically induced synaptic enhancement. BDNF also induced the recruitment of cortical circuits and increased the spatial distribution of active responses which was consistent with TBS-induced LTP in the ACC. In addition, the PPF was not significantly changed after BDNF application indicating that the BDNF produced synaptic enhancement was most likely postsynaptic. However, we cannot completely rule out the possible presynaptic effects of BDNF on glutamate release that cannot be detected by PPF. It has been reported in the ACC that the level of BDNF is related to changes in the uptake of glutamate and/or glutamate release [25].

### Mechanisms of BDNF induced synaptic enhancement in the ACC

Multiple ions/receptors have been reported to contribute to synaptic LTP [26], depending on the induction protocol, regions of the brain, recording methods, and age of animals [27]. TBS induced LTP in the ACC has been well investigated. It has been shown that, in response to TBS, synaptic responses in the ACC exhibited LTP lasting many hours. Induction of this form of LTP requires the activation of NMDA receptors, mGluRs, and L-VGCCs. In the dentate gyrus, it was reported that BDNF induced synaptic enhancement was NMDAR independent while other study showed that the enhancement was blocked by NMDA receptor in spinal dorsal horn [7, 28]. LTP is divided into early-phase and late-phase LTP (> 3 h). We found that BDNF in the ACC directly induces late-phase LTP, which was NMDA receptor independent. This result suggests that NMDA receptor is not necessary for BNDF induced late-phase LTP in the ACC. Moreover, L-VGCC and mGluRs were required for BDNF induced synaptic enhancement.

AC1 was considered critical for TBS produced synaptic enhancement in the ACC [29], we also found that BDNF produced enhancement was blocked in genetic deletion of AC1 or by a selective AC1 inhibitor NB001. These results provided strong evidence that ACC synaptic transmission might through AC1 signal pathway, although the exact signaling pathway remains to be determined. In the ACC, CP-AMPA receptors and PKMζ are involved in TBS-LTP expression in our previous studies. In the present study, we found that CP-AMPA receptors and PKMζ were also required for the expression of BDNF produced synaptic enhancement; suggesting that BDNF is likely contributing to postsynaptic form of LTP. Furthermore, BDNF induced synaptic enhancement and TBS-LTP occluded each other in the ACC, suggesting that BDNF induced LTP is postsynaptic. The synaptic source of BDNF can be multiple. Thibault et al. [15] reported that peripheral inflammation increased the expression of BDNF in neurons and glial cells in the ACC. Future studies are needed to determine molecular mechanism for BDNF regulation and release in the ACC.

### Functional and clinical implications

ACC has been indicated in different high-brain functions, including chronic pain, fear, anxiety, and depression [8, 9, 11]. Evidence has accumulated that BDNF is involved in chronic or persistent pain in the ACC. BDNF was upregulated in the ACC in rats with inflammatory pain. Injections of recombinant BDNF, or a viral vector synthesizing BDNF, into the ACC triggered sustained pain hypersensitivity [15]. In 2014, Thibault et al. reported that local injections of recombinant BDNF or a viral vector synthesizing BDNF into the ACC induced sustained cold hypersensitivity. Pharmacological blockade of BDNF-TrkB signaling, through local injection of cyclotraxin-B in the ACC prevented the emergence of cold hypersensitivity and passive avoidance behavior [15]. In addition to the ACC, BDNF in the spinal cord also underlies the development and maintenance of inflammatory and neuropathic pain [30, 31]. However, compared with the upregulation of BDNF in the ACC and spinal dorsal horn, BDNF is downregulated in hippocampus in spared nerve injury (SNI) model. These studies suggest that BDNF in different brain areas may play diverse roles in the process of chronic pain. BDNF may underlie chronic pain in the ACC and spinal dorsal horn, while involved in pain memory and emotional deficits in the hippocampus [15, 32–34]. Previous studies in the hippocampus showed that BDNF can enhance glutamate release [4, 35]. In the ACC, Koga et al. [12] reported a form of presynaptic LTP in the ACC and this pre-LTP is required for injury induced anxiety. Although we did not detect changes in PPF of evoked responses in the ACC of the present study, we cannot rule out effect of BDNF on glutamate release as well as the uptake of glutamate at synapses. Thus, it is likely that BDNF may affect ACC related anxiety.

The genetic mutation and altered BDNF levels in the ACC have been reported in patients with mental diseases [16, 17]. Sellmeijer et al. [36] reported that chronic pain-induced anxiodepressive-like consequences are underpinned by ACC hyperactivity. Combined with our present data, the activity of ACC enhanced by BDNF may play a pivotal role in the modulation function of BDNF on mental diseases. BDNF may also contribute to fear memory [35]. Zhao et al. reported that the long-term changes in the ACC synapses is required for trace fear memory. Pharmacological or genetic blockade of the GluN2B subunit in the ACC impaired the formation of early contextual fear memory [37]. In summary, BDNF in the ACC may contribute to many pathological processes. The long-term synaptic changes in the ACC synapses may determine these pathological mechanisms. In our present study, we demonstrate that NB001, a selective AC1 inhibitor, blocked BDNF-induced synaptic enhancement in the ACC, which may be useful for future treatment of BDNF-related diseases.

## Data Availability

The datasets used and/or analyzed during the current study are available from the corresponding author on reasonable request.

## Abbreviations

ACC: Anterior cingulate cortex
BDNF: Brain-derived neurotrophic factor
TrkB: Tropomyosin receptor kinase B
LTP: Long-term potentiation
TBS: Theta-burst stimulation
PPF: Paired-pulse facilitation
ACSF: Artificial cerebrospinal fluid
MEA: Multielectrode array
fEPSP: Field excitatory postsynaptic potential
AC1: Adenylyl cyclase subtype 1
L-VGCC: L-type voltage-gated calcium channels
mGluRs: Metabotropic glutamate receptors
CP-AMPARs: Calcium-permeable AMPA receptors
PKMζ: Protein kinase Mζ
